# Quality of information about oral cancer in Brazilian Portuguese available on Google, Youtube, and Instagram

**DOI:** 10.4317/medoral.23374

**Published:** 2020-02-10

**Authors:** Kamilla Karla Maurício Passos, Augusto César Leal da Silva Leonel, Paulo Rogério Ferreti Bonan, Jurema Freire Lisboa de Castro, Maria Luiza dos Anjos Pontual, Flávia Maria de Moraes Ramos-Perez, Danyel Elias da Cruz Perez

**Affiliations:** 1 DDS, MSc, School of Dentistry, Department of Clinical and Preventive Dentistry, Universidade Federal de Pernambuco, Recife, Pernambuco, Brazil; 2 DDS, PhD, School of Dentistry, Universidade Federal da Paraíba, João Pessoa, Paraíba, Brazil; 3 DDS, PhD, School of Dentistry, Department of Clinical and Preventive Dentistry, Universidade Federal de Pernambuco, Recife, Pernambuco, Brazil

## Abstract

**Background:**

To evaluate the quality of oral cancer information in Brazilian Portuguese on Google, YouTube, and Instagram.

**Material and Methods:**

The first 100 links of each platform characterized the initial sample. The websites and Instagram were evaluated using the JAMA benchmarks, the Discern instrument, and the Flesch readability index (Flesch Reading Ease). The existence of Health on the Net (HON) code was also registered on websites. The usefulness of each video on YouTube was classified as not useful, slightly useful, moderately useful, or very useful.

**Results:**

Thirty-four websites, 39 Instagram posts, and 57 videos were evaluated, of which 18 (33.3%) websites and 19 (48.7%) Instagram posts covered only 2 of the 4 JAMA benchmarks. For the Discern instrument, 20 (37%) and 18 (33.3%) websites exhibited low and moderate reliability, respectively, while 26 (66.7%) Instagram posts were of low confidence. The level of intelligibility of both websites and Instagram was difficult. Only three websites exhibited the HONcode. Forty-one (71.9%) videos on YouTube were moderately useful.

**Conclusions:**

Information on oral cancer on the Internet in Brazilian Portuguese is of low quality. Thus, educational and governmental institutions have a responsibility to produce and indicate reliable sources of information for the population.

** Key words:**Access to information, internet, oral cancer.

## Introduction

Globally, it is estimated that 354,864 new cases of oral cancer occurred in 2018, with 177,384 deaths. In Brazil, oral cancer represents the 5th most frequent cancer among males, with an estimated 11,200 new cases in men and 3,500 in women each year of the 2018–2019 period (1,2). Early diagnosis represents one of the greatest challenges in oral cancer. Failures of early detection of the disease by professionals and the lack of information and awareness among patients represent some factors contributing to delays in the diagnosis of oral cancer (3,4). Thus, there is a need to educate patients and health professionals, mainly dentists and physicians, about the risk factors, signs, and symptoms of the disease (5).

In this context, the use of the Internet as a source of health information has grown over the years. Patients and professionals have resorted to this tool for knowledge (6). Studies have shown that online information can contribute to the individual’s awareness of oral health, improved prognosis and adherence to treatment, and the facilitation of professional-patient communication (7,8). On the other hand, several authors have warned of the difficulty in certifying whether the information published on the Internet is valid and reliable (7-9). In terms of health content, this issue is especially worrying, since there is a risk to the segment of the population which is unaware of the existence of criteria used to identify the quality of information online (7).

The most commonly used Internet tools for access to information are search engines such as Google and Yahoo (4). In addition, other platforms have begun to act as channels of communication and access to health information, such as YouTube and Instagram. The main feature of these platforms, called social media, is the ability to provide greater interactivity between people in widely separated places. Online content sharing, which allows public participation, creates the possibility of a discussion between those who produce and those who read, resulting in collaboration in addressing health problems and population welfare (10).

Data on the quality of information about oral cancer on the Internet are scarce (4,5,8,11,12). Considering also that Brazil is one of the most populous countries of the world and with a high prevalence of oral cancer (1), it is surprising that there are no studies evaluating published content on this disease in the Portuguese language (Brazilian varieties). Thus, the objective of this study was to evaluate the quality of oral cancer information available in Brazilian Portuguese on the Internet and compare it with data from studies conducted in other countries.

## Material and Methods

In this study, we evaluated the Google search website, the video sharing platform YouTube, and the photo app Instagram. The contents of these communication channels in Brazilian Portuguese were assessed, addressing at least one of the following aspects: epidemiological and clinical characteristics, risk factors, and treatment of oral cancer.

- Data collection

The websites were identified in the search engine Google (www.google.com.br) using the keyword “câncer de boca,” the term commonly used in Brazilian Portuguese. The YouTube search (www.youtube.com) used the site’s default settings for videos related to oral cancer using the same keyword. For social media Instagram, the hashtag “cancer de boca” (#cancerdeboca) was used. All searches were conducted in February 2018, and for each communication channel, the initial sample comprised the first 100 listed links.

- Selection of websites

From the 100 websites of the initial sample, duplicate, off-site, and password-only sites were excluded, as were sites that addressed only oral potentially malignant disorders. Websites were also excluded if they addressed other types of cancer, presented only the surgical technique for treatment of oral cancer, or were book reviews or summary journal sites (4).

- Selection of Instagram posts

From the 100 postings of the initial sample, we excluded duplicate posts, postings that addressed only oral potentially malignant disorders or other types of cancer, and posts exclusively on surgical procedures for treatment of oral cancer (4).

- Selection of YouTube videos

For the 100 videos of the initial sample, the following exclusion criteria were used: duplicate or deleted videos, as well as videos with audio failures, and videos exclusively on oral potentially malignant disorders or that addressed only surgical techniques for oral cancer treatment. Videos on other cancers, self-help personal and misleading videos, such as religious content that offers spiritual healing or encourages the use of therapies not yet proven, were also excluded (5).

- Content analysis of websites, Instagram posts, and videos

The quality of the information was evaluated by two evaluators (K.K.M. Passos and A.C.L.S. Leonel), specialists in Oral Pathology and Medicine, who were previously trained in the analysis tools used. Any disagreements between the reviewers regarding the categorization and analysis of a site/video/posting were resolved by a third evaluator.

- Website analysis

According to their affiliation, the sites were classified as health professional, government, general health, online encyclopedias, news portal, and education sites. One of the parameters used to evaluate the quality of the information were the Journal of the American Medical Association (JAMA) benchmarks: the exhibition of authorship of health content, exhibition of attribution (references), current view (date of information), and disclosure of property, sponsorship, advertising policies, or conflicts of interest (4,13). The higher the number of criteria contemplated, the greater the credibility of the site or posting.

Another tool used for analysis was Discern, a free evaluation tool (www.discern.org.uk) which can be used by patients, professionals, and information editors. Discern comprises a total of 16 questions divided into three sections: Questions 1 to 8 address the reliability of the publication, and questions 9 to 15 address specific details of information regarding treatment options. Question 16 concerns the overall assessment of the instrument. Each question is scored on a scale of 1 to 5 (1 = the publication is poor, and 5 = the publication is of good quality) (4,11). At Discern website, the importance of each question is clearly defined, and guidelines for rating each question are also presented. In the present study, only the first section of the questionnaire was used for reliability assessment.

The Flesch Index of readability (Flesch Reading Ease) as adapted to Brazilian texts was also used (14). This method classifies the readability of a text on a scale from 0 (very difficult) to 100 (very easy) based on a calculation that takes into account the number of syllables per word and words per sentence. The adapted formula is given by the following equation:

Reading score = 248, 835 − (1,015 × ASL) − (84.6 × ASW) where:

ASL = mean number of words per sentence

ASW = mean number of syllables per word

Calculations were performed using Microsoft word processing software. From the Flesch index it is possible to categorize the texts under the following categories (14):

1. index between 75–100 (very easy texts = suiTable for primary school from 1st to 5th year);

2. index between 50–74 (easy texts = suiTable for elementary school grades 6-9);

3. index between 25–49 (fairly difficult texts = suiTable for high school or college); and

4. Index between 0–24 (very difficult texts = suiTable only for specific academic areas).

The existence of the Health on the Net Foundation (HON) seal (HONcode) was also recorded. The seal indicates adherence to a code of conduct for medical and health care websites which allows users to know the source and purpose of the information presented. The HON rates the fulfilment of eight criteria: 1. officialdom; 2. complementarity; 3. privacy; 4. assignment, references, and up-to-dateness; 5. reasoning; 6. transparency; 7. financial disclosure, and 8. advertising policy (4,15).

- Analysis of Instagram posts

According to their affiliation, the Instagram posts were classified as being by health professionals, profiles of general health, health insurers, and personal profiles (non-health professionals). For the analysis of information quality, the JAMA benchmarks, the Discern instrument, and the Flesch readibility index were used, applied as in the evaluation of websites.

- Analysis of YouTube videos

Each video had its upload source classified as institution of higher education institution, government, health professionals, TV channels, channels on general health, and personal profile. The relationship of the viewers with the videos was evaluated based on the interaction index (5).

(number of likes – number of dislikes) x 100% / total number of views

To assess the usefulness of videos in providing information on the signs, symptoms, and risk factors of oral cancer, a “utility score” (Table 1) was designed to categorize each video as not useful (0 points), slightly useful (1 to 3) moderately useful (4 to 7), or very useful (8 to 10) (5).

**Table 1:**
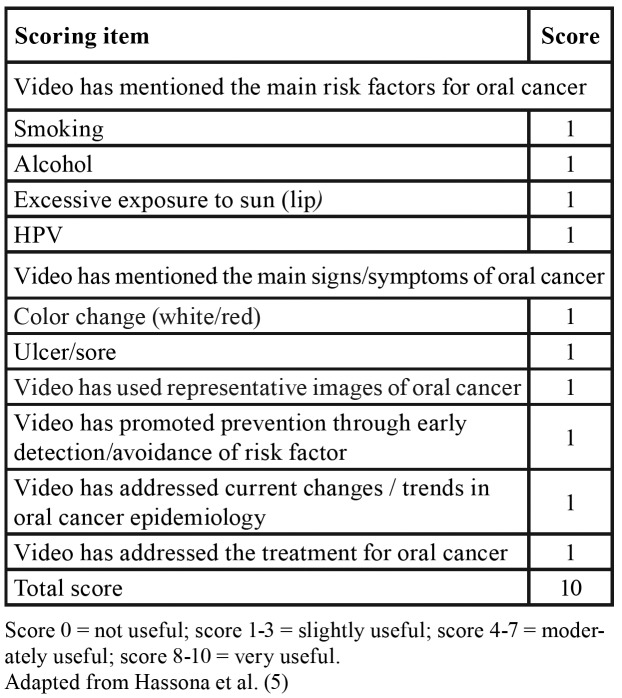
Criteria for utility score on YouTube videos.

- Statistical analysis

The data were tabulated and analyzed using IBM SPSS Statistics 20.0 software (IBM Corporation, New York, United States). The associations between the variables of affiliation and other evaluated criteria in each platform, were assessed using chi-square tests with a significance level of 5%. Spearman’s correlation coefficient was used to correlate the interaction index and utility scores in YouTube videos.

## Results

- Websites

The Google search yielded a total of 445,000 results. Of the 100 links of the initial sample, the 54 websites remaining after the application of the exclusion criteria were evaluated. Most websites were from news portals (n = 19, 35.2%), followed by health professionals (n = 17, 31.5%) and general health sites (n = 12, 22.2%). Considering the JAMA benchmarks, 7 sites (13%) did not include any criteria, 14 (25.9%) had a single criterion, 18 (33.3%) had 2 criteria, and 15 (27.8%) presented 3 criteria. None of the evaluated websites m*et al*l four criteria. Regarding the presence of the HONcode, only 3 sites (5.6%) had this certification. The Discern instrument identified 20 websites (37%) of low reliability, 18 (33.3%) of moderate reliability, and 16 (29.6%) of high reliability. For the Flesch Index, 28 websites (51.9%) were classified as difficult, while 20 (37%) were considered easy. Only 1 website (1.9%) was classified as very easy and 5 (9.3%) very difficult.

There was no association between the affiliation of websites and the JAMA (*p* = 0.07) (Table 2) and Discern criteria (*p* = 0.06), presence of HONcode (*p* = 0.716), or Flesch index (*p* = 0.518).

- Instagram

The Instagram search resulted in a total of 1208 posts. Of the 100 postings of the initial sample, the 39 publications remaining after the application of the exclusion criteria were selected for the study.

Most postings were performed by health professionals (n = 34, 87.2%). According to the JAMA benchmarks, 19 (48.7%) postings included 2 criteria, 14 (35.9%) 3 criteria, and 6 (15.4%) presented all 4 criteria. Regarding Discern, 26 (66.7%) publications exhibited low reliability content, while 10 (25.6%) had content with a high confidence level. Only 1 post (2.6%) had moderate reliability, and for 2 (5.1%) the questionnaire could not be applied because there were few words in the publication. For the Flesch Index, 21 (53.8%) posts were classified as difficult, while 14 (35.9%) were easy. Only 1 publication (2.6%) was classified as very difficult and in 3 cases (7.7%) the criterion could not be applied, in 2 of them because there were few words and in 1 case the publication was in topics, which would influence the result.

There was no association between the affiliation of posts and the JAMA (*p* = 0.26) or Discern criteria (*p* = 0.986), or the Flesch index (*p* = 0.607) (Table 3).

- YouTube

This search yielded a total of 57,900 videos. From the initial sample of 100 videos, the 57 remaining after the application of the exclusion criteria were analyzed. The mean duration of the videos was 6.67 minutes (range of 1 min to 60 mins) and the mean number of views was 11,106.81 (range of 14 to 191,267).

Most videos were uploaded by TV channels (n = 23, 40%), followed by personal profiles (n = 11, 19.3%) and health professionals (n = 10, 17.5%). The majority (n = 51, 89.5%) promoted the early detection of oral cancer. However, in 40 (70.2%) videos the source of the published content was not mentioned, and in 54 (94.7%) other sources of information about the disease were not indicated. Tobacco (n = 44, 77.2%) and alcohol (n = 43, 75.4%) were the most-cited risk factors, while 25 videos (43.8%) mentioned ultraviolet radiation and 14 (24.6%) indicated HPV. It was observed that 37 (64.9%) videos mentioned poor oral hygiene and 32 (56.1%) the use of dental prosthesis as risk factors for the disease. Most videos (n = 41, 71.9%) were classified as moderately useful.

There was no association between upload sources and utility scores (*p* = 0.596) (Table 4). A weak correlation between the interaction index and the utility score (r = 0.262) was observed.

**Table 2:**
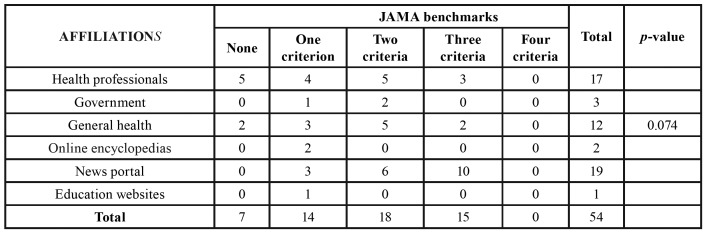
Association between affiliations and JAMA benchmarks in websites.

**Table 3:**
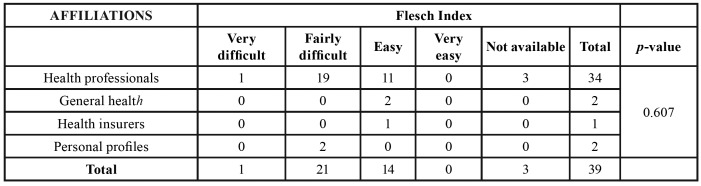
Association between affiliations and Flesch Reading Ease in Instagram posts.

**Table 4:**
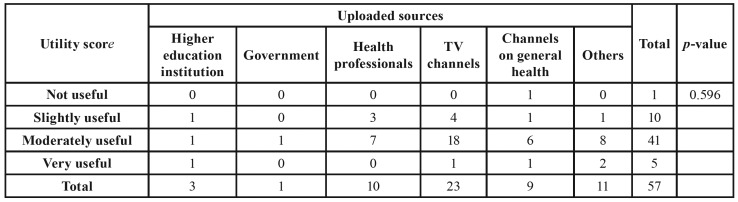
Association between upload source and utility score on YouTube videos.

## Discussion

The internet has great potential for spreading health information. Whatever the communication channel used, it is known that its content is able to influence the decisions of an individual about his health, including changes in lifestyles (7,16).

In Brazil, most cases of oral cancer are diagnosed at an advanced clinical stage, which results in poor prognosis and a high mortality rate (17). Among the reasons for the delay in diagnosis are the lack of available information about the disease and its low quality (3,4). Dantas *et al*. (18) observed that socioeconomic factors, especially educational level, significantly influence the diagnosis and survival of patients with oral cancer. In this context, this study evaluated the quality of oral cancer information in Brazilian Portuguese on Google, YouTube, and Instagram. To date (July 2019), this study is the first to evaluate Instagram as a source of information on oral cancer. Additionally, this is the only study to analyze the quality of information about this disease in more than one type of media available in the Portuguese language in Brazil.

Although the Internet enables free debate on various issues, there is a difficulty in identifying information without scientific evidence. Eysenbach *et al*. (9) indicated the need to standardize evaluation tools and use them to determine the quality of a website. However, assessing the quality of information on the Internet is a complex task, and the adoption of a single criterion that considers all content is difficult, resulting in a superficial evaluation. To help Internet users analyze this information, several methods and tools have been created (4). In this study, when the JAMA benchmarks and the Discern instrument were applied, the websites and Instagram presented similar results, with low reliability content in general. Vivien *et al*. (12) observed similar results when evaluating sites in French. The two most frequent JAMA benchmarks were those of authorship and contemporaneity. For Instagram in particular, these two criteria are mandatory in all publications, because authorship is assigned to the profile and the posting date is part of the app formatting. The attribution and disclosure of conflicts of interest or sponsorship was not mentioned by most websites or evaluated for Instagram posts, showing a lack of concern for informing the reader of the original source of the shared content. In addition, it is important to highlight that although health professionals are the main parties responsible for the Instagram posts, two-thirds of the publications presented low reliability.

HONcode is a direct indicator of the quality of information, since it ensures compliance with ethical standards by content producers and allows users to know the origin of published information. In this survey, only three sites presented the seal. Other studies around the world also identified a small number of sites with this certification (4,19). Importantly, in this study, sites of Institutions of higher education and the government did not present the seal, while sites created to disseminate general health information exhibited such certification. This suggests that even after two decades of activities and translation into 35 languages, the HONcode has not yet gained visibility and therefore remains unknown by the Brazilian public sector. The absence of this seal on sites of important institutions, which have a relationship of trust with the netizens, indicates that concern for certifying the quality of the information in the Internet is still scarce. Another possible cause for low seal adhesion is the fact that the annual review required for seal maintenance is not free (15).

The websites and Instagram posts presented a difficult level of intelligibility. A systematic review assessing their quality for health information consumers revealed that the texts, as evaluated by the Flesch index, required a high reading level on the part of the users (9). Varela-Centelles *et al*. (8) identified most English-language websites with information on oral cancer as presenting a high level of intelligibility, which influences the level of understanding in people with low literacy. In this study, the Flesch Index revealed that the texts were suiTable for users who have a high level of reading to understand the published information. In Brazil, approximately 50% of the population has completed elementary education and one third presents low functional literacy (20). Information is more easily understood when there is a predominance of short sentences and a substitution of technical words by simpler ones. Careful construction of the text is important, as it is not possible to determine precisely the educational level of the user who will receive the message. Jayaratne *et al*. (7) proposed that the level of intelligibility should be indicated next to each link in the search results list, which would allow users to access content compatible with their level of education.

Turning to YouTube, our study identified most videos as moderately useful, because they mentioned some of the main risk factors, presented clinical signs of the disease, provided information about treatment, and promoted the early prevention of oral cancer, similar to the findings of Hassona *et al*. (5). Most videos mentioned ill-fitting dental prostheses and poor oral hygiene as risk factors associated with the disease. Despite some studies raising these associations (21,22), the International Agency for Research on Cancer (IARC) considers carcinogens agents for oral cancer, the tobacco, alcoholic beverages, HPV16, betel nut, and ultraviolet radiation for lip carcinoma (23,24). Like other media evaluated by this study, YouTube allows the posting of content by any user. This explains why most upload sources consist of TV channels and other users who are not health professionals (16). On the other hand, the number of videos posted by institutions of higher education and government agencies was very small. This fact can be attributed to YouTube’s default settings. Videos that show a commercial ad before displaying will appear first in the results. If the video does not have an ad, the search rank will depend on a consideration of the publication based on several factors such as the number of likes, views, and interactions. A video with few views may appear among the first results because the upload source has many followers. Therefore, the list of results is not based on the level of confidence of the information, because this criterion is not evaluated on YouTube.

Although the aim of this study was to evaluate the quality of oral cancer information on Internet (reliability, readability, certification), some sources in the initial sample presented misinformation on the disease. Of the 100 videos in the initial sample, 4 were excluded from the survey because they were considered misleading videos which could endanger the health of the population. One of them encouraged the use of unproven therapies and others 3 videos were of religious nature, stating that the disease could be eliminated by a religious authority. In the remaining media evaluated, sites and Instagram, misleading information was not evidenced. An alternative to prevent misinformation would be to encourage the development and adoption of resources through which users themselves are able to recognize and signal inaccurate and misleading content (6). To do this, there must be a commitment from the government, health professionals, and educational institutions to alert patients about the shortcomings and hazards that they will face when researching health information on the Internet.

In summary, internet information on oral cancer available in Portuguese from Brazil is of low quality. Despite considering only content in Brazilian Portuguese, the data of this study are comparable to those of studies that evaluated content in other languages, including English. As there is no control over content posted on the Internet, consumers must develop the skills to access, understand, and use such content securely. At the same time, health professionals and educational and government entities have a responsibility to contribute to improving health content on the Internet. They should participate in the production and delivery of this information, directing users to good quality sources.
